# Appropriateness and acceptability of a Tele-Yoga intervention for people with heart failure and chronic obstructive pulmonary disease: qualitative findings from a controlled pilot study

**DOI:** 10.1186/s12906-015-0540-8

**Published:** 2015-02-07

**Authors:** Lucy Selman, Kelly McDermott, DorAnne Donesky, Tracie Citron, Jill Howie-Esquivel

**Affiliations:** Department of Palliative Care, Policy and Rehabilitation, King’s College London, Cicely Saunders Institute, Bessemer Road, London, SE5 9PJ U.K; University of California, San Francisco, Osher Center for Integrative Medicine, 1545 Divisadero Street, 4th Floor, San Francisco, CA 94115-3010 U.S.A; Department of Physiological Nursing, UCSF School of Nursing Building, University of California, San Francisco, 2 Koret Way, Box 0610, San Francisco, CA 94143-0610 U.S.A

**Keywords:** Yoga, Tele-medicine, Meditation, Relaxation, Heart failure, Chronic obstructive pulmonary disease, Dyspnoea, Complex intervention, Qualitative, Medical Research Council framework

## Abstract

**Background:**

Heart failure (HF) and chronic obstructive pulmonary disease (COPD) are highly prevalent and associated with a large symptom burden, that is compounded in a dual HF-COPD diagnosis. Yoga has potential benefit for symptom relief; however functional impairment hinders access to usual yoga classes. We developed a Tele-Yoga intervention and evaluated it in a controlled pilot trial. This paper reports on the appropriateness and acceptability of the intervention and the evaluation design.

**Methods:**

A controlled, non-randomised trial was conducted of an 8-week Tele-Yoga intervention versus an educational control (information leaflets mailed to participants with one phone call a week). Biweekly one-hour Tele-Yoga classes were implemented via multipoint videoconferencing that connected participants to live classes via an Internet connection to their televisions. Semi-structured qualitative interviews were conducted with participants post study exit to explore reasons for and experiences of participating, including views of study outcome measures and physiological tests. Transcribed interviews were analysed using thematic content analysis.

**Results:**

Fifteen people participated in the pilot study (7 in the intervention group, 8 in the control). Of these, 12 participants were interviewed, 6 in each group, mean age 71.2 years (SD 10.09); 3 were male. Themes are reported in the following categories: acceptability and appropriateness of the intervention, potential active ingredients of the intervention, acceptability and appropriateness of the control, participation in the research, and acceptability of the testing procedures. The intervention was acceptable and appropriate: the intervention group reported enjoying yoga and valuing the home-based aspect and participants described a high symptom burden and social isolation. However, technological problems resulted in poor video-streaming quality for some participants. Potential active ingredients included physical postures, breathing exercises and guidance in relaxation and meditation. The educational control intervention was acceptable and appropriate, with participants reporting little effect on their well-being and no impact on mechanisms hypothesised to explain yoga’s effectiveness. The questionnaires and home physiological testing were acceptable to participants.

**Conclusions:**

Tele-Yoga is an acceptable and appropriate intervention in people with HF and COPD and further research is warranted to refine the technology used in its delivery. Findings provide guidance for researchers working in tele-interventions, yoga, and similar populations.

**Trial registration:**

ClinicalTrials.gov Identifier: NCT02078739 (4 March 2014).

## Background

Heart failure (HF) and chronic obstructive pulmonary disease (COPD) are major causes of morbidity and mortality worldwide, resulting in a burden to society that is both substantial and increasing [1-4]. It is estimated that 40% of people with a primary diagnosis of HF also have COPD [5] and that 21% of those with a primary COPD diagnosis have HF [6]. Both diseases are characterised by symptoms of functional impairment and limited exercise capacity along with high levels of breathlessness, fatigue, depression and anxiety [7-10]. When present in the same patient, HF and COPD can dramatically worsen symptoms and health limitations [11]. Existential and social problems faced by HF and COPD patients include dealing with the uncertainty of the prognosis, hopelessness, struggling to find meaning in life, altered self-image, difficult changes in relationships and social roles, increasing dependence on others and feeling isolated and unsupported [12-15]. There is evidence that social isolation is associated with higher rates of anxiety, depression, hospitalisation and death [16,17].

Yoga may be an appropriate complementary therapy for individual and dual-diagnosed HF and COPD patients because of its emphasis on movement-coordinated breathing and low impact fitness. Yoga involves relaxation and meditation, which may improve the psychological symptoms experienced in this population [18], and incorporates breath training techniques which may be of particular benefit in coping with breathlessness [19]. Many pulmonary rehabilitation programs include components of yoga in their prescribed exercises [20] and there is increasing evidence of the cardiovascular benefits of yoga; a lifestyle programme incorporating yoga is funded by Medicare for people with heart disease [21,22]. The authors tested a centre-based Iyengar yoga programme in participants with HF and in participants with COPD and found that yoga was safe and feasible [23-25]. In addition, yoga participants had greater improvements in symptoms and functional performance compared to those receiving usual care. These findings were consistent with a previous pilot study showing improvements in physical function, symptom stability and overall well-being among HF patients [26] and the results of recent meta-analyses in HF and COPD [27,28].

Although these preliminary findings are promising, transportation to a central class location was found to be a major barrier to adherence in this chronically-ill older population [24]. Cost-effective interventions to support patients with HF and/or COPD who are socially isolated and unable to travel are needed. Tele-interventions, which use telecommunications technologies to support long-distance clinical health care, health-related education and public health [29], are gaining popularity and relevance as increasing numbers of people are accessing the Internet for health information, support and fitness. Recent trials of remote cardiac rehabilitation suggest that the use of tele-technologies may help increase enrolment, reduce risk factors and improve benefit-cost ratio [30,31]. Advances in multi-point interactive videoconferencing technologies have made it easier for individuals who face geographic barriers to participate in traditional group-based exercise programmes from home (e.g. [32]). These programmes have huge cost-saving potential as well as the ability to reach a larger number of people in the community. Access to live trainers and commitment to a person or group may also help with adherence to fitness programmes [33], while an online platform provides convenience and accountability. Successful implementation of a real-time home-based yoga programme would increase access and may increase adherence among participants.

We therefore developed a controlled pilot study of a video conference yoga intervention for dual diagnosis patients with HF and COPD (Tele-yoga) versus an educational control [34,35]. In-depth qualitative interviews were conducted with participants post study exit to explore experiences of study participation. The use of qualitative methods is increasingly recognised as best practice in the development and evaluation of complex interventions [36-39] and is central to the United Kingdom’s Medical Research Council (MRC) guidance in this area [40,41]. Following the MRC framework, this paper presents qualitative findings from participant exit interviews to assess acceptability and appropriateness of the intervention, educational control and study design, and explore potential active ingredients of the intervention. Our primary objective was to inform intervention refinement for future studies of Tele-interventions in advanced disease populations.

## Methods

### Study design

In-depth semi-structured qualitative interviews were conducted with participants of a quasi-experimental, non-randomised trial of a Tele-Yoga intervention, after study completion. We followed the MRC framework for the development and evaluation of complex interventions, which recommends the use of an iterative, phased approach that harnesses qualitative as well as quantitative methods to improve study design, execution and generalisability of results [41]. This framework offers a structured and evidence-based way to develop and evaluate complex interventions and is commonly cited in the complementary therapy research literature [42-44].

### Sampling and inclusion criteria

The population for this study was patients with dual HF and COPD diagnoses according to medical record documentation. Patients with New York Heart Association (NYHA) class I-III HF [45] and moderate-severe COPD (defined as post bronchodilator Forced Expiratory Volume (FEV1) in 1 sec < 80% predicted, FEV1/Forced Vital Capacity ratio < 70% and history of smoking) were eligible for inclusion. Additional inclusion criteria were: English-speaking, permission from their provider to participate in the study, TV and broadband internet connection, enough space to practice yoga in front of the TV, willingness to have research assistant install videoconferencing equipment, medical record documentation of hospitalisation for HF in the previous 24 months (but no hospitalisation for the past 3 months) and a score of 3 on the Mini-Cog Test (indicating a negative screen for dementia) [46].

A convenience sample of participants was recruited via American Lung Association Better Breathers Clubs in the San Francisco Bay Area and cardiology and pulmonary clinics at the University of California, San Francisco (UCSF) Medical Center. In line with the requirements of specialised yoga classes for people with high levels of need and the exploratory aims of the study, we aimed to recruit 7–8 people in each group (intervention and control) and stopped recruitment at this point. Recruitment occurred from March 2012 to February 2013.

Participants were not aware which group they would be allocated to when they consented to the study. Because of timing requirements, the first 7 participants recruited were allocated to the intervention group and the next 8 to the control group.

### Ethical approval

Written informed consent was sought from all participants prior to enrolment in the study. The study protocol was reviewed and approved by the University of California, San Francisco Institutional Review Board (ref. 12–08383).

### Intervention

Home-based one-hour Tele-Yoga classes were offered twice weekly for 8 weeks, ending in November 2012. The yoga protocol was based on the previously tested yoga programs for COPD [24] and HF [23], developed by an Iyengar yoga instructor with expertise in working with the chronically ill. Please contact the authors for the protocol. Classes integrated held postures, breathing exercises (slow breathing and extended exhalation breathing), imagery, meditation and relaxation. The teacher modified postures as needed to meet the physical ability of each participant and offered corrections and adjustments with verbal cues, further explanation, and modelling of poses to participants. Participants were provided with a yoga mat if needed, and encouraged to use common household items as props where necessary (e.g. a chair with a firm seat and back, pillows for a bolster or a towel in the place of a strap).

### Technology

Multi-point videoconferencing equipment (DocBox technology (MicroDesign, Colchester, VT)) was installed in the homes of the intervention group during the baseline home visit. DocBox enabled participants to connect to live streamed classes via an Internet connection to their televisions. Participants could see the teacher (and vice versa) and received personalised instruction. Participants were not meant to be able to see or hear other participants. A research nurse (qualified RN) monitored each session for safety by viewing live video of all participants.

### Control

An attention control was utilised: participants in the control group received education materials once a week and the research nurse called each week to discuss the information with them. The control group ran for 8 weeks, ending in March 2013. The education materials covered the following topics: evaluating health information, problems sleeping, elder abuse, flu vaccinations, accessing information about complementary and alternative therapies online, accessing information about medications online, depression and a low sodium diet.

### Data collection

Semi-structured in-depth qualitative interviews were conducted with participants in the pilot study between 1 week and 3 months after completion of the study. Interview schedules covered: symptoms and function, motivations and expectations of participating in the study, experiences of the intervention or control, views and experiences of yoga, views of the battery of outcome measures and physiological testing procedures, and suggestions for improvement of the intervention and/or the evaluation. Interviews were either conducted in person at the participant’s home or via the telephone according to the preferences of the participant. All interviews were recorded and transcribed verbatim.

The outcome measures administered in the study were: the Mini-Cog for dementia [46], Kansas City Cardiomyopathy Questionnaire [47], Memorial Symptom Assessment Schedule [48], Dypsnea-12 [49], the St. George’s Respiratory Questionnaire [50], the PHQ-8 for the evaluation of depression [51], General Sleep Disturbance Scale [52], Adult Sleep Assessment scale [53], Multidimensional Assessment of Interoceptive Awareness [54] and Five Facet Mindfulness Questionnaire [55].

The physiological testing procedures conducted in participants’ homes were: spirometry [56], weight, upper body (biceps) and lower body (quadriceps) testing with arm curls and chair stands, balance using the one-leg stand test, and endurance with the home-adapted six-minute walk test [57]. Quantitative data are not reported here, but are included as participants were asked about the acceptability of measures and testing procedures. Demographic and clinical data (see Table 1) were collected by the research nurse at baseline.

**Table 1:** **Demographic and clinical characteristics**

**ID code**	**Intervention/control group**	Gender*	**Age group**	Education**	**Living situation**	**Oxygen use**	NYHA class***
ID#01	I	M	60-64	3	Alone	Some	3
ID#02	I	F	80-84	4	Alone, in community housing complex	Constant	2
ID#03	I	M	70-74	Missing	With spouse	None	2
ID#04	I	F	75-80	4	Alone	Some	3
ID#05	C	F	70-74	2	With son	Constant	3
ID#06	I	F	45-50	2	With spouse	None	2
ID#07	I	F	85-90	3	Alone	Constant	3
ID#10	C	F	70-74	4	Alone	None	2
ID#11	C	F	75-80	2	Alone, assisted living	None	3
ID#13	C	F	70-74	4	With spouse	None	2
ID#14	C	M	70-74	4	With spouse	Some	2
ID#15	C	F	65-70	2	With family/friend	Constant	3

*M = male, F = female.

**0 = less than high school, 1 = no college, high school only, 2 = some college, 3 = 4 year college degree, 4 = graduate degree.

***New York Heart Association class [45].

### Qualitative analysis

Data were analysed thematically following the principles of qualitative content analysis [58,59], within a minimal realist paradigm [60,61]. Analysis was conducted in three stages. In the first stage, LS and KM each independently created two coding frames, one for control and one for intervention data. This was done through LS and KM inductively identifying themes and sub-themes arising in transcripts of two interviews with participants in the intervention group and two with participants in the control group. In the second stage, LS and KM met to compare coding frames, discuss differences and agree on a final version. Preliminary findings were discussed with the project team and feedback incorporated into the coding frames. LS and KM then applied the final coding frames to all transcripts. In a final stage, LS grouped identified themes and sub-themes according to research questions relating to the criteria of acceptability, appropriateness and potential active ingredients (Table 2). Deviant cases were highlighted within each theme to describe the breadth of the data. Analysis was conducted in NVivo v10 [62] and Dedoose 4.5 [63].

**Table 2:** **Criteria, research questions and corresponding themes in data analysis**

**Criterion**	**Research question(s)**	**Corresponding themes**
Appropriateness of the intervention	How well was the intervention aligned with patient needs?	Patients’ well-being, symptoms and function
Acceptability of the intervention	How acceptable was the intervention to participants? What challenges arose for intervention participants?	Experiences of taking part in Tele-Yoga
	Did participants continue to practice what they learnt?	
Potential active ingredients of the intervention	What aspects of the intervention were reported to have therapeutic benefit, and why?	Reported effects of the intervention
Acceptability of the control	Was the control group considered acceptable?	Usefulness of the education materials
		Views about being in the control group
Appropriateness of the control	Did the control group intervention have any effect which might confound effects of the yoga intervention?	Effect of the control on participant coping
Participation in the research	What motivated participants to take part in research of this nature?	Participants’ motivations for participation
	What expectations did participants bring when they consented to participate?	Participants’ expectations of participation
Acceptability of the testing procedures	Were the testing procedures acceptable to participants?	Perceptions of measures and home physiological testing

## Results

**Figure 1:**
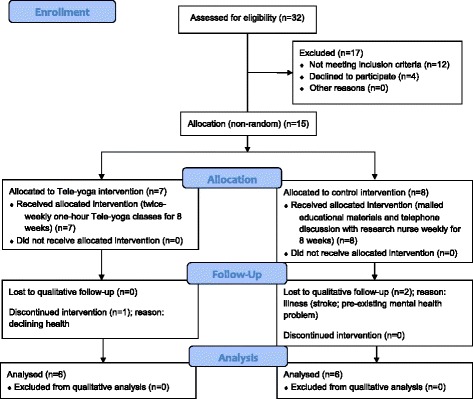
**CONSORT 2010 flow diagram.**

Fifteen participants took part in the pilot study (7 from the intervention group and 8 from the control group). Of these, twelve participants were interviewed, six in each group (see Figure 1 for the CONSORT flow diagram [64]). One of the participants in the intervention group dropped out prior to the study end and was unavailable for interview. The remaining six intervention group participants had each attended at least 13 of the 16 classes offered. Two of the participants in the control group were too sick to participate (one suffered two strokes and one had a relapse of a pre-existing mental health condition). One interview was conducted by phone, the rest in-person. Interviews ranged from 25 to 53 minutes in length, mean 35 minutes (SD 8.47).

Participant characteristics are presented in Table 1. Mean age of participants was 71.2 years (SD 10.09); three were male. All except one participant (ID#04) was of Caucasian ethnicity.

Four main themes were identified in the data: Current situation, the research process, views of the education materials (control group only) and views of the intervention (intervention group only). Sub-themes that related directly to the research questions of acceptability, appropriateness, and potential active ingredients are presented here (see Table 2). Findings are discussed by criterion and research question.

### Acceptability of the intervention

#### How acceptable was the intervention to participants?

Participants in the intervention group reported positive experiences of doing yoga during the study, with two participants saying they enjoyed it (ID#04, ID#07). The home-based nature of the Tele-Yoga intervention was viewed positively, with two participants highlighting that it was motivational:

“But this, I’ve got to tell you, [having it at home] was fantastic. I got my butt up to do it, I’m serious. It did”. ID#06

ID#02: “I keep seeing other DVDs that I might, you know, if I can get myself to, to get going in the morning and throw something like that in and do it”.

Interviewer [clarifying]: “Do you think that having the live class was more motivating than just doing the DVD?”

ID#02: “Yeah, oh yeah… I mean, [the DVD’s] been sitting there for a long time”.

The modifications (e.g. sitting in a chair) were reported to be useful and the fact that classes were specifically targeted at participants with HF and COPD was seen as a benefit:

“My neighbour across the hall goes to this [yoga] programme. It’s in a senior centre over in [city region] on Saturdays. But that kind of thing doesn’t appeal to me because even if the people are all designated older, I mean over 65 or something… That’s very different from being severely disabled at 83, you know”. ID#02

#### What challenges arose for intervention participants?

Three participants found the yoga classes challenging; one participant had problems with her back and found some postures (e.g. twists and sitting up) difficult for that reason (ID#07), one found getting down on the floor difficult (ID#04) and another would have preferred a class that was entirely chair-based and a little shorter (ID#02):

“The best part was certainly the sitting exercises. Because I really can’t get up. Partly it’s the breathing. I have to move very slowly. So I can’t get to where I want to be by the time she’s finished that exercise, or that posture”. ID#02

However, another participant would have preferred a longer class (ID#04). Rearranging the furniture was reported to be slightly inconvenient but the home-based nature of the class was considered positive (ID#06). The same participant reported that he would have liked to have been given a bolster as a prop.

Three participants (ID#01, ID#02, ID#06) had problems with the videoconferencing technology. Connections were unreliable, making it hard to follow the teacher:

“The basic thing is that the technology has been so frustrating… I began to kind of know what she was going to do, but you know, I’m looking at the thing, and she moves and she’s got four pairs of arms, and I have no idea which one I’m supposed to be imitating, you know?… And I get three out of five words… It was just really frustrating”. ID#02

One of the participants with similar problems complained about the IT support employed to assist with trouble-shooting:

“I’d just keep having problems every week, and so I would call the [IT support] guys. I’d call [IT support worker] and tell him what was going on. And it was just like every week it kept going on and on and on. And then finally it was so bad one day… I had to go through the yoga instructor to talk to them about it”. ID#06

Another consequence of the technological problems was that occasionally participants would be able to see or hear one another. There were mixed feelings about connecting with the other group participants in this way. Three participants reported either that they would have liked to meet the other class participants prior to the online classes (ID#03) or that they would have liked to see the other participants during the classes (ID#02, ID#07):

“It would be nice if we could see the other people, too, struggling away to get up out of the chair. And you know, sometimes I’m sure there is always someone that’s worse off than you are”. ID#07

However, the remaining three participants reported that they didn’t want to see or hear other participants and didn’t like it when technical problems caused that, as they wanted privacy both for themselves and the others:

“No, no [I would not have liked to have seen the other participants]. Well, I don’t think so, no… I just want to see the instructor… But you know, all of a sudden you’re looking at the screen and it would be somebody else… You know, you’re in the middle of doing your yoga and everything and then you hear, I hear one of the people in there, an older lady and she’s like having problems. And I’m getting upset because I can hear her and she can’t catch her breath”. ID#06

#### Did intervention participants continue to practice what they learned in the Tele-Yoga classes?

Five of the participants in the intervention group reported that they planned to continue with yoga, either through watching a DVD and consulting print-outs given out at the end of the series or in one instance through attending a class offered at a nearby senior centre. Two participants requested that recordings be made available of one of the actual classes (ID#03, ID#06). One participant said he continued to practice some of the yoga techniques every day (ID#03), while another participant reported that since the classes stopped and life had become more stressful it had been difficult to continue to practice yoga (ID#06). The participant who did not plan to continue to do yoga was the most unwell of the group and had found the postures challenging (ID#07).

Four participants talked about ways in which they had been able to integrate elements of the yoga class, particularly the breathing exercises, in their lives after the study, e.g.

“When I started to get really short of breath and I saw those numbers bang up, and I was thinking okay, I’ve got to slow down. So I went into the yoga breathing of breathing in my nose, one two, and then breathing out my mouth one two three four. And then just opening myself up, and just slowed it down. And the nurses were amazed that I could do it, yeah… Because most people hyperventilate, because they get anxious. I just went the other way”. ID#04

One participant talked about sharing the skills he had learned with others:

“Well I’ve definitely learned opening your – or trying to open up your chest makes a difference, and the twists and the different techniques we practice. In fact I had – when I was flying back from South Carolina a few weeks ago, a woman was like ‘Do you have asthma?’ and she is pushing a wheelchair and you know, she didn’t have medical [insurance]. And I said ‘Well, here’s some breathing exercises that I learned’”. ID#01

### Appropriateness of the intervention

#### How well was the intervention aligned with participant needs?

Participants in both groups described a range of burdensome and severe symptoms with a significant impact on their lives: shortness of breath, asthma, chronic coughing, chemical sensitivity and allergies, hypoxia/muscle spasms, arthritis, dizziness, back pain, Achilles pain, chills, cold sweats, fatigue, depression, weight loss and sleep problems. Three participants had recently had pneumonia:

“Well, right now I’m recovering from pneumonia so you know I’m kind of weak and feeble; frail I guess is a better word. But er, my general health is good but it’s just my breathing that’s the problem… I’m really having a hard time bouncing back this time”. ID#05

Associated with these symptoms was poor function, as expected in this population:

“Oh yeah, right now it’s really bad. I can barely walk from one room to the other. If I don’t stand up and catch my breath before I go to the bathroom, I’m out of breath. I’m breathing really deep like I just ran somewhere”. ID#05

Illness affected participants’ ability to travel, their social and financial situation, their ability to work, lift or carry and do housework, and their leisure time (“I’m going out and about less and less”. ID#02). One participant, an exception, described how she was able to maintain an active life despite the limitations of her illness:

“I don’t go to the mountains anymore, I hike mainly at sea level on really flat land, [but] other than that I have a very active life. I go out every day, I usher, I do strength training, I play bocce, I play the piano, take classes – you know, I do a lot of stuff”. ID#10

Unrelenting or worsening symptoms and shortness of breath were associated with anxiety and worry:

“Yeah, I’m kind of starting to get worried. I mean, is this going to be it? Is this going to be my new ‘normal’? Gosh, I hope not. Because I mean I can’t, practically can’t do anything, you know. And if I push myself I go sit back down and it takes me forever to recover”. ID#05

Dealing with illness and treatment using nebulizers and other devices could take up a large proportion of a participant’s day, as described vividly by one participant:

“Unfortunately I have bronchiectasis so my daily life is pretty much consumed by treatments and pills. I really have to fight for free time… If I get up at like 11–12 - I get up late - I’m really not ready to go until about 5 in the afternoon… If I start at 9 o’clock at night I’m about ready to go to sleep about 2 o’clock in the morning”. ID#14

Self-care, in the form of diet and exercise, was perceived as important and participants were motivated to improve their symptoms:

“I do almost religiously walk about 20–25 minutes a day just, you know in the neighbourhood or actually I can’t even, I live in this beautiful, beautiful place and because of my allergies and asthma, this time of the year from March until maybe July I have to walk indoors at the mall”. ID#13

These findings confirm that many people with COPD and HF live with a high physical and psychological symptom burden, poor physical function, social isolation, and treatments that limit their free time. This suggests a yoga intervention designed to assist with the symptoms experienced and easily available at home is appropriate in this population. The perception of self-care as important further suggests that these people would be motivated to participate in interventions perceived to be effective and/or helpful.

### Potential active ingredients of the intervention

#### What aspects of the intervention were reported to have therapeutic benefit, and why?

Several physical benefits from the physical postures and breathing exercises were reported: increased flexibility, release of muscular tension in the neck and upper back, motivation to walk more, ability to exercise, improved stamina, and skills to better deal with shortness of breath, e.g.

“I was able to play – I made it through a whole game of basketball with my son. Every time I got out of breath I started the yoga breathing… I made it the whole game. I scored five points”. ID#06

In addition, participants described psychological benefits from participating: slowing down/relaxing, improved sleep and being better able to deal with anxiety or stress, e.g.

“What it has done is made me more aware of when to slow down and how to slow down… Just like when my SAT [oxygen saturation] rate went from 92 to 80, you know, instead of panicking I just slowed down the breathing”. ID#04

The same participant described improved self-awareness and resulting benefits:

“Well, I don’t know if I’m making progress. What I’m doing is – I know my enemy better. And I am more aware of what I can do and not do… It’s the combination of the breathing, the movements, the positions. It’s very holistic”. ID#04

### Acceptability of the control

#### Was the control intervention acceptable to participants?

All members of the control group reported that participation was satisfactory or enjoyable, that the education intervention was not burdensome in terms of time and effort and that the education materials were relevant, e.g.

“I enjoyed working with [the research nurse] and [the telephone calls] were fine. I didn’t feel that they were imposing on my time or anything; I didn’t mind that at all”. ID#10

“Just about every one of [the education materials] told me something that I didn’t know or that I knew but forgot, you know, so every one of them was helpful and interesting. I didn’t, none of them bored me or you know seemed a waste of time to me”. ID#05

However, three of the participants (a retired registered nurse, a retired pharmacist, and an educator) were already well informed about their conditions and found some of the materials rather elementary. The one set of materials that all control group participants reported to be useful was that regarding accessing health information online.

Participants in the control group were asked how they felt about not being in the intervention group. Four said they didn’t mind which group they were in (“Whatever they wanted to do with me was fine [laughs]”. ID#05). One participant said she felt disappointed, but also that she wasn’t sure she was up to doing the yoga and was glad she had received the education materials (ID#11). One participant said she would not have wanted to be in the yoga intervention group as she had back problems (ID#10).

### Appropriateness of the control

#### Did the control group intervention have any effect which might confound effects of the yoga intervention?

Four control group participants reported that the control intervention had little or no effect on them (e.g. “I can’t honestly say that it had any influence on me one way or another”. ID#14). Two participants reported some effect. One said the information on diet was inspiring to her:

Interviewer: “You said you found the depression one illuminating. Do you think that may have affected the way you cope with your illness?”

ID#15: “Yes. And I think the food one, you know, gave me more hope for my [diet] plan that I’m going through and inspired me”.

Another said she thought participating in the study may have helped her become more accepting of her illness and able to deal with the stigma associated with it (ID#05).

### Participation in the research

#### What motivated participants to take part in research of this nature?

Participants reported a range of reasons for agreeing to participate in the study: previous enjoyment or appreciation of yoga (ID#07, ID#06, ID#02, ID#01), recognised need for exercise and interest in yoga (ID#11), the research study considered an interesting opportunity to improve their situation (ID#01, ID#04), attraction to the home-based nature of the yoga intervention (ID#01) and it being gentle and modified for people with heart and lung problems (ID#04), recommendation by a physiotherapist (ID#03), desire to help demonstrate the benefit of yoga (ID#13), because they valued research (ID#13), for altruistic reasons (ID#13, ID#10, ID#05), because they liked the principal investigator (ID#13, ID#14) and for personal benefit (ID#15, ID#05). Three participants (ID#07, ID#06, ID#04) had had prior positive experience of participating in research, e.g.

“[Research] is actually how I found out about all my issues. Being in studies has actually been good for me”. ID#06

#### What expectations did participants have of the intervention and control?

Participants in the yoga group described the following expectations: help with symptoms, breathing, stretching, flexibility, strengthening, toning, fitness/exercise and being more active; for example:

“I thought I’d be able to be more active and released from the cage. Because that’s what you feel like, you’re in a cage… I was hoping that this would kind of turn me around, back toward being more active”. ID#07

Participants in the control group reported that they thought they may learn something to help them with illness (ID#10, ID#15, ID#13), such as breathing techniques:

“Well I thought I would get something out of it and the more I can do for myself, the shape I’m in, I’m game for, so any time I get a chance to participate in anything, I like to learn and you know find out more about my condition and I thought it might be helpful to me”. ID#15

### Acceptability of testing procedures

#### Were the testing procedures acceptable to participants?

Participants found the questionnaires acceptable and straight-forward, although repetitive; this was interpreted in different ways:

“I tried to do them truthfully, but you know some of them were a little tricky. It seemed like ‘Oh wait a minute, they just asked me a question like that one there, is this a trick thing?’ you know”. ID#15

“[The questionnaires] were very good. And I thought some of them certainly looked like they were prepared by psychologists. I have a law degree although I never – I passed half the bar – that was another career, but it reminded me of when you have a trial they take depositions and they give people interrogatories and they ask similar questions in a different format but really they are asking you the same thing and what they are looking for is consistency”. ID#13

No one thought the questionnaires were over-burdensome, although one participant (ID#11) could not complete the measures when the research nurse came to visit as she was too unwell, so completed them the following day.

Physiological testing in the home was found to be acceptable, with participants appreciating not having to travel into hospital to complete the tests (“Oh, I was very glad that somebody saved us a trip to the city [laughs]”. ID#13). However, one participant found the physiological tests quite challenging (ID#05) and another would have preferred a different format:

“Then the other thing, the walk test. Um, the idea was to walk around this 20 foot measuring tape you know for six minutes. So I’m walking around and around and around and I felt like a hamster and I just wondered why there might not be an alternative since I live in a nice, flat place”. ID#10

## Discussion

This is the first study to provide evidence regarding the acceptability and appropriateness of a Tele-Yoga intervention in a population diagnosed with both HF and COPD. To our knowledge there has been no prior research into yoga as an intervention in this population. Our findings inform future investigators of ways in which to refine a tele-intervention and trial design in response to participant experiences. The home-based nature of the intervention was strongly valued by these participants, many of whom were socially isolated by their illness. Participants found enjoyment in the classes and found them helpful, although their functional limitations made certain poses, such as the standing poses and transitions from one pose to another, challenging. However, problems with the technology resulted in poor video-streaming quality and low satisfaction with the technology for half the participants. The technological issues affected intervention fidelity on multiple occasions for several of the participants. Participants’ descriptions of living with a dual HF-COPD diagnosis highlighted the severe symptom burden, functional impairment and social isolation experienced by this patient population. Our findings thus underscore the profound limitations and challenges of a dual HF-COPD diagnosis, contributing to our understanding of a population in which previous research has predominantly focused on epidemiology and diagnosis [11,65].

Participants in the intervention group described benefits they perceived as resulting from taking part in Tele-Yoga, many of which have been reported in other studies of yoga: increased flexibility [66] and strength [67], improved motivation and ability to exercise [68], improved ability to cope with shortness of breath [24,69], improved sleep [70] and being better able to cope with anxiety or stress [71-74]. Our findings suggest that in this population relaxation and long, slow breathing may be particularly important active ingredients of yoga. Increased body awareness and mindfulness, identified in other yoga intervention studies, did not appear to play a role in this population [75,76]. Larger scale studies are needed to identify which components of a yoga intervention are most efficacious and their mechanism of action in this population.

The use of an educational control group was found to be acceptable and appropriate, with participation in either arm of the study perceived to be useful by participants. Findings also suggest that the control intervention could be successful in controlling for the nonspecific intervention effects of expectation bias, attention, and the therapeutic alliance or relationship between participant and yoga teacher [77]: participants’ expectations did not seem to have significantly affected their experience of taking part in the study, and control group members reported that they spoke to the research nurse regularly and enjoyed the contact and follow-up. Control group participants did not report beneficial effects on physical well-being, although the education materials were perceived as potentially useful psychologically, supporting coping and lifestyle changes. The quantitative data (to be published elsewhere) is needed to confirm or disconfirm these findings. There was no reported impact of the control on mechanisms that are hypothesised to explain the effectiveness of yoga (e.g. fitness, relaxation, mindfulness and breath), an important consideration in the evaluation of control interventions [78]. Finally, the outcome measures and home physiological testing were overall acceptable to participants.

Our study adds to the evidence base demonstrating the value of following the MRC framework in designing and evaluating complex interventions based on complementary therapies [42-44] and in seriously ill populations [79,80]. In this framework, the pre-clinical or theoretical phase relates to identifying the evidence that the intervention might have the desired effect. Phase 1, modelling, aims to improve the understanding of intervention components and their relationships, while Phase 2 aims to develop the optimum intervention and study design. The last stage of evaluation (Phase 3) involves designing and conducting the definitive randomised controlled trial, while Phase 4 examines long-term implementation of the intervention in practice. These phases are an iterative process rather than a hierarchy of steps, with movement from one step to another and back again as the intervention and evaluation process develop on the basis of evidence. We plan to follow the MRC framework in using the findings from this pilot study to further refine the Tele-Yoga intervention and trial design, eventually moving from Phase 2 to Phase 3, thus contributing to strengthening the evidence base relating to yoga in HF and COPD [28,81].

There are several limitations to this study. The qualitative component was embedded within an experimental study design, which meant a convenience rather than purposive sample participated. While this might compromise the representativeness of the sample and transferability of our findings to the wider HF-COPD population, we nevertheless achieved a sample spanning a wide range of age groups (45–90 years in the intervention group, 65–80 years in the control group), and both control and intervention groups included participants of NYHA class 2 and 3, and varying degrees of oxygen use, levels of education and living situations. This suggests that our sample was broadly representative of the population of interest. Another limitation is that the small sample size in each group means data saturation cannot be assumed; different participants might have reported different experiences and views. However, the diversity of the sample and the divergence of experiences and views reported in the rich dataset demonstrate the utility of the findings and the benefit of qualitative research when investigating the acceptability and appropriateness of novel interventions such as Tele-Yoga [39]. The design meant we were not able to explore the acceptability and feasibility of randomisation in this trial and this should be taken into account when considering the feasibility of a full-scale randomised trial with the same intervention and control. A further potential limitation is that the control group was not exactly matched to the yoga group in terms of time commitment and attention (intervention group participants had a greater time commitment and received more attention: 2 hours per week in the intervention group vs. 1 hour or less per week in the control group); while it is difficult to quantify attention in research of this nature, this would be an interesting area for future investigation prior to a full-scale trial. In addition, participants had varying levels of previous experience of yoga, with some reporting that they wished to help demonstrate the potential effectiveness of yoga, which may have biased their reporting. This should be born in mind in interpreting the findings of this feasibility study. Finally, the outcome measures and physiological tests administered to participants were not investigated individually but rather asked about as a group, in order to gauge the acceptability of the battery of measures and the concept of home physiological testing. Quantitative findings from the study regarding data completeness will shed further light on the acceptability of individual measures and testing procedures.

Our findings support further development and evaluation of home-based, real-time yoga interventions using simplified multipoint video technology. This will benefit from evolving and improved technology in the provision of online exercise. The technology was selected for use in this population because it was simple to use: the participant needed only to turn on the TV and push one button to join the class, and the technology was remotely managed once it was turned on. However, problems with the technology suggest that it requires further refinement. Online platforms could also be explored as viable alternatives. In addition, further research is required to explore the benefits, drawbacks and acceptability of participants being able to view and interact with each other as well as the teacher during classes. While this might mimic the social benefits of class participation in this socially isolated population, our findings suggest that not all people wish to engage with others in this way. Further research is required to explore contextual factors which might influence participant preferences in this regard [82].

## Conclusion

In conclusion, Tele-Yoga is an acceptable and appropriate intervention in participants with HF and COPD and further research is warranted to refine the technology used in its delivery. Findings suggest that an educational control can successfully control for the nonspecific effects of expectation bias, attention, and therapeutic alliance. Home-based physiological testing was acceptable in this population of participants with dual diagnosis of COPD and HF. Using the MRC framework, this pilot study provides evidence to refine the Tele-Yoga intervention, better understand active ingredients of the intervention, and design a successful trial. Findings also have applicability for researchers working with similar interventions and in other chronically ill populations.

## References

[1] Cowie MR, Wood DA, Coats AJ, Thompson SG, Suresh V, Poole-Wilson PA (2000). Survival of patients with a new diagnosis of heart failure: a population based study. Heart.

[2] Mosterd A, Hoes AW (2007). Clinical epidemiology of heart failure. Heart.

[3] Lopez AD, Mathers CD, Ezzati M, Jamison DT, Murray CJ (2006). Global and regional burden of disease and risk factors, 2001: systematic analysis of population health data. Lancet.

[4] Holguin F, Folch E, Redd SC, Mannino DM (2005). Comorbidity and mortality in COPD-related hospitalizations in the United States, 1979 to 2001. Chest.

[5] Mascarenhas J, Lourenco P, Lopes R, Azevedo A, Bettencourt P (2008). Chronic obstructive pulmonary disease in heart failure. Prevalence, therapeutic and prognostic implications. Am Heart J.

[6] Rutten FH, Cramer MJ, Grobbee DE, Sachs AP, Kirkels JH, Lammers JW (2005). Unrecognized heart failure in elderly patients with stable chronic obstructive pulmonary disease. Eur Heart J.

[7] Haworth JE, Moniz-Cook E, Clark AL, Wang M, Waddington R, Cleland JG (2005). Prevalence and predictors of anxiety and depression in a sample of chronic heart failure patients with left ventricular systolic dysfunction. Eur J Heart Fail.

[8] Solano JP, Gomes B, Higginson IJ (2006). A comparison of symptom prevalence in far advanced cancer, AIDS, heart disease, chronic obstructive pulmonary disease and renal disease. J Pain Symptom Manage.

[9] Zambroski CH, Moser DK, Bhat G, Ziegler C (2005). Impact of symptom prevalence and symptom burden on quality of life in patients with heart failure. Eur J Cardiovasc Nurs.

[10] Mullerova H, Lu C, Li H, Tabberer M (2014). Prevalence and burden of breathlessness in patients with chronic obstructive pulmonary disease managed in primary care. PLoS One.

[11] Hawkins NM, Virani S, Ceconi C: Heart failure and chronic obstructive pulmonary disease: the challenges facing physicians and health services. Eur Heart J 2013. 10.1093/eurheartj/eht192 23832490

[12] Murray SA, Kendall M, Boyd K, Worth A, Benton TF (2004). Exploring the spiritual needs of people dying of lung cancer or heart failure: a prospective qualitative interview study of patients and their carers. Palliat Med.

[13] Pinnock H, Kendall M, Murray SA, Worth A, Levack P, Porter M, et al. Living and dying with severe chronic obstructive pulmonary disease: multi-perspective longitudinal qualitative study. BMJ. 2011; 342:d142. 10.1136/bmj.d142 PMC3025692 21262897

[14] Selman L, Beynon T, Higginson IJ, Harding R (2007). Psychological, social and spiritual distress at the end of life in heart failure patients. Curr Opin Support Palliat Care.

[15] Selman L, Harding R, Beynon T, Hodson F, Coady E, Hazeldine C (2007). Improving end-of-life care for patients with chronic heart failure: “Let’s hope it’ll get better, when I know in my heart of hearts it won’t”. Heart.

[16] Scherer M, Himmel W, Stanske B, Scherer F, Koschack J, Kochen MM (2007). Psychological distress in primary care patients with heart failure: a longitudinal study. Br J Gen Pract.

[17] Chin MH, Goldman L (1997). Correlates of early hospital readmission or death in patients with congestive heart failure. Am J Cardiol.

[18] Casey A, Chang BH, Huddleston J, Virani N, Benson H, Dusek JA (2009). A model for integrating a mind/body approach to cardiac rehabilitation: outcomes and correlators. J Cardiopulm Rehabil Prev.

[19] Pomidori L, Campigotto F, Amatya TM, Bernardi L, Cogo A (2009). Efficacy and tolerability of yoga breathing in patients with chronic obstructive pulmonary disease: a pilot study. J Cardiopulm Rehabil Prev.

[20] Hodgkin JE, Celli BR, Connors GL (2009). Pulmonary rehabilitation: guidelines to success.

[21] Ornish D, Scherwitz LW, Billings JH, Brown SE, Gould KL, Merritt TA (1998). Intensive lifestyle changes for reversal of coronary heart disease. JAMA.

[22] Koertge J, Weidner G, Elliott-Eller M, Scherwitz L, Merritt-Worden TA, Marlin R (2003). Improvement in medical risk factors and quality of life in women and men with coronary artery disease in the Multicenter Lifestyle Demonstration Project. Am J Cardiol.

[23] Howie-Esquivel J, Lee J, Collier G, Mehling W, Fleischmann K (2010). Yoga in heart failure patients: a pilot study. J Card Fail.

[24] Donesky-Cuenco D, Nguyen HQ, Paul S, Carrieri-Kohlman V (2009). Yoga therapy decreases dyspnea-related distress and improves functional performance in people with chronic obstructive pulmonary disease: a pilot study. J Altern Complement Med.

[25] Donesky D, Melendez M, Nguyen HQ, Carrieri-Kohlman V. A responder analysis of the effects of yoga for individuals with COPD: who benefits and how? Int J Yoga Therap. 2012;22:23-36. 23070669

[26] Pullen PR, Nagamia SH, Mehta PK, Thompson WR, Benardot D, Hammoud R (2008). Effects of yoga on inflammation and exercise capacity in patients with chronic heart failure. J Card Fail.

[27] Gomes-Neto M, Rodrigues-Jr ES, Silva-Jr WM, Carvalho VO (2014). Effects of yoga in patients with chronic heart failure: a meta-analysis. Arq Bras Cardiol.

[28] Liu XC, Pan L, Hu Q, Dong WP, Yan JH, Dong L (2014). Effects of yoga training in patients with chronic obstructive pulmonary disease: a systematic review and meta-analysis. J Thorac Dis.

[29] Telehealth. [http://www.hrsa.gov/ruralhealth/about/telehealth/]

[30] Wakefield B, Drwal K, Scherubel M, Klobucar T, Johnson S, Kaboli P (2014). Feasibility and effectiveness of remote, telephone-based delivery of cardiac rehabilitation. Telemed J E Health.

[31] Worringham C, Rojek A, Stewart I (2011). Development and feasibility of a smartphone. ECG and GPS based system for remotely monitoring exercise in cardiac rehabilitation. PLoS One.

[32] Wu G, Keyes L, Callas P, Ren X, Bookchin B (2010). Comparison of telecommunication, community, and home-based Tai Chi exercise programs on compliance and effectiveness in elders at risk for falls. Arch Med Rehabil.

[33] Burke SM, Carron AV, Eys MA, Ntoumanis N, Estabrooks PA. Group versus individual approach? A meta-analysis of the effectiveness of interventions to promote physical activity. Sport and Exerc Psychol Rev. 2006; 2:19–35.

[34] Selman L, Citron T, Howie-Esquivel J, McDermott K, Donesky D (2013). Tele-yoga in patients with chronic obstructive pulmonary disease and heart failure - a mixed methods study of feasibility, acceptability and safety [abstract].

[35] Citron T, Howie-Esquivel J, McDermott K, Milic M, Donesky D (2013). Feasibility and safety of tele-yoga in patients with both chronic obstructive pulmonary disease and heart failure.

[36] Datta J, Petticrew M (2013). Challenges to evaluating complex interventions: a content analysis of published papers. BMC Public Health.

[37] Oakley A, Strange V, Bonell C, Allen E, Stephenson J (2006). Process evaluation in randomised controlled trials of complex interventions. BMJ.

[38] O’Cathain A, Thomas KJ, Drabble SJ, Rudolph A, Hewison J. What can qualitative research do for randomised controlled trials? A systematic mapping review. BMJ Open. 2013;3:e002889. doi:10.1136/bmjopen-2013-002889 10.1136/bmjopen-2013-002889 PMC3669723 23794542

[39] Plano Clark VL, Schumacher K, West C, Edrington J, Dunn LB, Harzstark A (2013). Practices for embedding an interpretive qualitative approach within a randomized clinical trial. J Mixed Methods Res.

[40] Craig P, Dieppe P, Macintyre S, Michie S, Nazareth I, Petticrew M, et al. Developing and evaluating complex interventions: the new Medical Research Council guidance. BMJ. 2008;337:a1655. 10.1136/bmj.a1655 PMC2769032 18824488

[41] Medical Research Council. Developing and evaluating complex interventions: new guidance. London: MRC; 2008.

[42] Thompson TD, Weiss M (2006). Homeopathy–what are the active ingredients? An exploratory study using the UK Medical Research Council’s framework for the evaluation of complex interventions. BMC Complement Altern Med.

[43] MacPherson H, Schroer S (2007). Acupuncture as a complex intervention for depression: a consensus method to develop a standardised treatment protocol for a randomised controlled trial. Complement Ther Med.

[44] Dhillon HM (2011). Researching complementary and alternative therapies: frameworks for CAM evaluation. Canc Forum.

[45] Association. TCCotNYH (1994). Nomenclature and criteria for diagnosis of diseases of the heart and great vessels.

[46] Borson S, Scanlan J, Brush M, Vitaliano P, Dokmak A (2000). The mini-cog: a cognitive ‘vital signs’ measure for dementia screening in multi-lingual elderly. Int J Geriatr Psychiatry.

[47] Green CP, Porter CB, Bresnahan DR, Spertus JA (2000). Development and evaluation of the Kansas City Cardiomyopathy Questionnaire: a new health status measure for heart failure. J Am Coll Cardiol.

[48] Portenoy RK, Thaler HT, Kornblith AB, Lepore JM, Friedlander-Klar H, Kiyasu E (1994). The Memorial Symptom Assessment Scale: an instrument for the evaluation of symptom prevalence, characteristics and distress. Eur J Cancer.

[49] Yorke J, Swigris J, Russell AM, Moosavi SH, Ng Man Kwong G, Longshaw M (2011). Dyspnea-12 is a valid and reliable measure of breathlessness in patients with interstitial lung disease. Chest.

[50] Jones PW, Quirk FH, Baveystock CM (1991). The St George’s respiratory questionnaire. Respir Med.

[51] Kroenke K, Strine TW, Spitzer RL, Williams JBW, Berry JT, Mokdad AH (2009). The PHQ-8 as a measure of current depression in the general population. J Affect Disord.

[52] Lee KA (1992). Self-reported sleep disturbances in employed women. Sleep.

[53] Lee KA, Ward TM (2005). Critical components of a sleep assessment for clinical practice settings. Issues Ment Health Nurs.

[54] Mehling WE, Price C, Daubenmier JJ, Acree M, Bartmess E, Stewart A (2012). The Multidimensional Assessment of Interoceptive Awareness (MAIA). PLoS One.

[55] Baer RA, Smith GT, Lykins E, Button D, Krietemeyer J, Sauer S (2008). Construct validity of the five facet mindfulness questionnaire in meditating and nonmeditating samples. Assessment.

[56] Miller MR, Hankinson J, Brusasco V, Burgos F, Casaburi R, Coates A (2005). Standardisation of spirometry. Eur Respir J.

[57] Du H, Davidson PM, Everett B, Salamonson Y, Zecchin R, Rolley JX (2010). Assessment of a self-administered adapted 6-minute walk test. J Cardiopulm Rehabil Prev.

[58] Hsieh HF, Shannon SE (2005). Three approaches to qualitative content analysis. Qual Health Res.

[59] Pope C, Ziebland S, Mays N. Analysing qualitative data. BMJ. 2000; 320:114. 10.1136/bmj.320.7227.114 PMC1117368 10625273

[60] Hammersley M (2009). Challenging relativism: the problem of assessment criteria. Qual Inq.

[61] Seale C. The quality of qualitative research*.* London: Sage; 1999.

[62] Ltd. QIP (2012). NVivo qualitative data analysis software, Version 10.

[63] Dedoose Version 4.5, web application for managing, analyzing, and presenting qualitative and mixed method research data (2013). Los Angeles, CA: SocioCultural Research Consultants, LLC (www.dedoose.com).

[64] Schulz KF, Altman DG, Moher D, Group ftC: CONSORT (2010). Statement: updated guidelines for reporting parallel group randomised trials. BMC Med.

[65] Zeng Q, Jiang S (2012). Update in diagnosis and therapy of coexistent chronic obstructive pulmonary disease and chronic heart failure. J Thorac Dis.

[66] DiBenedetto M, Innes KE, Taylor AG, Rodeheaver PF, Boxer JA, Wright HJ (2005). Effect of a gentle Iyengar yoga program on gait in the elderly: an exploratory study. Arch Phys Med Rehabil.

[67] Tran MD, Holly RG, Lashbrook J, Amsterdam EA (2001). Effects of hatha yoga practice on the health-related aspects of physical fitness. Prev Cardiol.

[68] Bryan S, Pinto Zipp G, Parasher R (2012). The effects of yoga on psychosocial variables and exercise adherence: a randomized, controlled pilot study. Altern Ther Health Med.

[69] Bernardi L, Spadacini G, Bellwon J, Hajric R, Roskamm H, Frey AW (1998). Effect of breathing rate on oxygen saturation and exercise performance in chronic heart failure. Lancet.

[70] Mustian KM, Sprod LK, Janelsins M, Peppone LJ, Palesh OG, Chandwani K (2013). Multicenter, randomized controlled trial of yoga for sleep quality among cancer survivors. J Clin Oncol.

[71] Michalsen A, Grossman P, Acil A, Langhorst J, Ludtke R, Esch T (2005). Rapid stress reduction and anxiolysis among distressed women as a consequence of a three-month intensive yoga program. Med Sci Monit.

[72] Lavretsky H, Epel ES, Siddarth P, Nazarian N, Cyr NS, Khalsa DS (2013). A pilot study of yogic meditation for family dementia caregivers with depressive symptoms: effects on mental health, cognition, and telomerase activity. Int J Geriatr Psychiatry.

[73] Selman LE, Williams J, Simms V (2012). A mixed-methods evaluation of complementary therapy services in palliative care: yoga and dance therapy. Eur J Cancer Care (Engl).

[74] Selman L, Higginson IJ (2010). ‘A softening of edges’: a comparison of yoga classes at palliative care services in New Delhi and London. Int J Palliat Nurs.

[75] Carmody J, Baer RA (2008). Relationships between mindfulness practice and levels of mindfulness, medical and psychological symptoms and well-being in a mindfulness-based stress reduction program. J Behav Med.

[76] Daubenmier JJ (2005). The relationship of yoga, body awareness, and body responsiveness to self-objectification and disordered eating. Psychol Women Q.

[77] Kinser PA, Robins JL, Control Group Design (2013). Enhancing rigor. Research of mind-body therapies for depression evidence-based complementary and alternative medicine.

[78] Whitehead WE (2004). Control groups appropriate for behavioral interventions. Gastroenterology.

[79] Higginson IJ, Evans CJ, Grande G, Preston N, Morgan M, McCrone P (2013). Evaluating complex interventions in end of life care: the MORECare statement on good practice generated by a synthesis of transparent expert consultations and systematic reviews. BMC Med.

[80] Farquhar M, Preston N, Evans CJ, Grande G, Short V, Benalia H (2013). Morecare: Mixed methods research in the development and evaluation of complex interventions in palliative and end-of-life care: report on the MORECare consensus exercise. J Palliat Med.

[81] Cramer H, Lauche R, Haller H, Dobos G, Michalsen A (2014). A systematic review of yoga for heart disease.

[82] Beauchamp MR, Carron AV, McCutcheon S, Harper O (2007). Older adults’ preferences for exercising alone versus in groups: considering contextual congruence. Ann Behav Med.

